# Implementation outcomes of a health systems strengthening intervention for perinatal women with common mental disorders and experiences of domestic violence in South Africa: Pilot feasibility and acceptability study

**DOI:** 10.1186/s12913-022-08050-x

**Published:** 2022-05-13

**Authors:** Zulfa Abrahams, Yuche Jacobs, Mbali Mohlamonyane, Sonet Boisits, Marguerite Schneider, Simone Honikman, Nadine Seward, Crick Lund

**Affiliations:** 1 Alan J Flisher Centre for Public Mental Health, Department of Psychiatry and Mental Health, University of Cape Town, Building B, 46 Sawkins Road, Rondebosch, Cape Town, 7700 South Africa; 2 Alcohol, tobacco and other drug research unit, South African Medical Research Council, Cape Town, South Africa; 3 Perinatal Mental Health Project, Alan J Flisher Centre for Public Mental Health, Department of Psychiatry and Mental Health, University of Cape Town, Cape Town, South Africa; 4 Centre for Global Mental Health, Health Service and Population Research Department, Institute of Psychiatry, Psychology & Neuroscience, King’s College London, London, UK; 5 Centre for Implementation Science, Health Service and Population Research Department, Institute of Psychiatry, Psychology & Neuroscience, King’s College, London, London, UK

**Keywords:** Acceptability, Feasibility, Fidelity, Adoption, Perinatal, Common mental disorders, Domestic violence

## Abstract

**Background:**

South Africa has a high burden of perinatal common mental disorders (CMD), such as depression and anxiety, as well as high levels of poverty, food insecurity and domestic violence, which increases the risk of CMD. Yet public healthcare does not include routine detection and treatment for these disorders. This pilot study aims to evaluate the implementation outcomes of a health systems strengthening (HSS) intervention for improving the quality of care of perinatal women with CMD and experiences of domestic violence, attending public healthcare facilities in Cape Town.

**Methods:**

Three antenatal care facilities were purposively selected for delivery of a HSS programme consisting of four components: (1) health promotion and awareness raising talks delivered by lay healthcare workers; (2) detection of CMD and domestic violence by nurses as part of routine care; (3) referral of women with CMD and domestic violence; and (4) delivery of structured counselling by lay healthcare workers in patients’ homes. Participants included healthcare workers tasked with delivery of the HSS components, and perinatal women attending the healthcare facilities for routine antenatal care. This mixed methods study used qualitative interviews with healthcare workers and pregnant women, a patient survey, observation of health promotion and awareness raising talks, and a review of several documents, to evaluate the acceptability, appropriateness, feasibility, adoption, fidelity of delivery, and fidelity of receipt of the HSS components. Thematic analysis was used to analyse the qualitative interviews, while the quantitative findings for adoption and fidelity of receipt were reported using numbers and proportions.

**Results:**

Healthcare workers found the delivery and content of the HSS components to be both acceptable and appropriate, while the feasibility, adoption and fidelity of delivery was poor. We demonstrated that the health promotion and awareness raising component improved women’s attitudes towards seeking help for mental health conditions. The detection, referral and treatment components were found to improve fidelity of receipt, evidenced by an increase in the proportion of women undergoing routine detection and referral, and decreased feelings of distress in women who received counselling. However, using a task-sharing approach did not prove to be feasible, as adding additional responsibilities to already overburdened healthcare workers roles resulted in poor fidelity of delivery and adoption of all the HSS components.

**Conclusions:**

The acceptability, appropriateness and fidelity of receipt of the HSS programme components, and poor feasibility, fidelity of delivery and adoption suggest the need to appoint dedicated, lay healthcare workers to deliver key programme components, at healthcare facilities, on the same day.

## Background

South Africa, like many other low- and middle-income countries, has a high burden of common mental disorders (CMDs), such as depression and anxiety, among perinatal women. One in every three to four perinatal women experience symptoms of depression (27%-39%) [1–4], while one in every five to six women experience symptoms of anxiety (15%-23%) [1, 5]. CMDs are associated with multidimensional poverty [6], food insecurity [2] and domestic violence [7]. Detection and treatment of CMDs is key to improving the health outcomes of perinatal women and their children [8, 9].

Yet CMDs are not routinely detected in all perinatal women attending public healthcare facilities in South Africa, nor are adequate treatment options available to those who are detected [10]. Since an effectively performing health system is vital for improving detection and care, the Health System Strengthening in sub-Saharan Africa (ASSET) study collaborated with the Western Cape Department of Health (WC DoH) to design a health systems strengthening (HSS) programme aimed at improving the quality of care for perinatal women with CMDs and experiences of domestic violence [11].

Successful implementation outcomes (i.e., the effects of deliberate actions taken to implement new practices) are considered essential to achieving changes in clinical or service outcomes. Several implementation outcomes [12] were identified to measure the success of implementing the HSS programme at three public healthcare facilities in Cape Town, in a pilot study. This study aims to describe the acceptability, appropriateness, feasibility, adoption, fidelity of delivery, and fidelity of receipt of the HSS programme, to detect and treat perinatal women with CMDs and experiences of domestic violence.

## Methods

### Setting

In South Africa, free public healthcare is available at primary, secondary and tertiary level healthcare facilities [13]. Midwife obstetric units (MOUs) and basic antenatal care (BANC) clinics are located in Primary Healthcare (PHC) facilities. In addition to antenatal and postnatal care, MOUs attend to low-risk deliveries for approximately 60% of South African women [14]. At MOU and BANC clinics, care is provided by nursing staff (professional, enrolled and assistant nurses) and lay healthcare workers such as HIV Counsellors, Breastfeeding Counsellors and Health Promotion Officers. In addition, the Department of Health contracts non-profit organisations (NPOs) to provide community-based care to patients and/or their caregivers attending primary healthcare facilities.

Community-based care is provided by teams of community health workers (CHWs) who are managed by an Outreach Team Leader (OTL) and provide support within a designated area. OTLs are professional or enrolled nurses, who supervise teams of 15-20 CHWs (healthcare workers without a professional qualification), who work in the communities they live in, and receive a stipend based on their years of experience. Community-based care includes health promotion, palliative care, wound care, and wellness monitoring, including maternal and new-born support.

Three public healthcare facilities, managed by the WC DoH, situated in the Cape Metropolitan health district in Cape Town, were purposively selected to ensure that the interventions were tested in different settings. The facilities included (1) a MOU situated in a predominantly Coloured (people of mixed ancestry [15]) community in the Southern-Western sub-district with high patient numbers (±185 antenatal patients per week), (2) a MOU situated in a predominantly Black community in the Klipfontein-Mitchell’s Plain sub-district with high patient numbers (±250 antenatal patients per week), and (3) a BANC clinic situated in the Khayelitsha-Eastern sub-district, in a community of Black South Africans and Black Africans from nearby countries such as Malawi, Nigeria, Zimbabwe, Zambia, Mozambique and Swaziland small patient numbers (±100 antenatal patients per week). Each healthcare facility was supported by between one (the BANC clinic) and four NPOs providing community-based care to patients living in the surrounding communities.

### Study design

The ASSET study consisted of three phases: pre-implementation, intervention development, and pilot evaluation [11]. During the pre-implementation phase of the study, several contextual barriers to detection and care of perinatal women with CMD and experiences of domestic violence were identified [10] including: (i) high levels of stigma, low mental health literacy and health seeking behaviour; (ii) low levels of detection for mental health disorders and experiences of domestic violence;(iii) poor linkages to care;and (iv) limited availability of treatment for CMD and experiences of domestic violence.

**Fig. 1:**
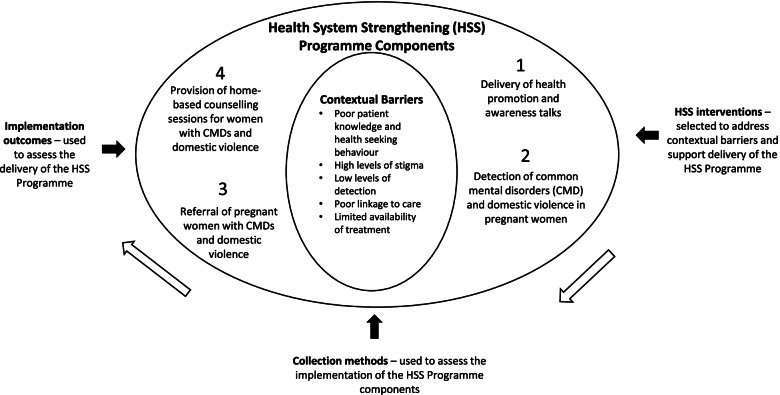
Design of the HSS programme, HSS interventions and implementation outcomes

The intervention development phase of the study was used to design a HSS programme (Fig. 1), guided by a Theory of Change workshop. The HSS Programme consists of four main components: health promotion and awareness raising, detection, referral, and treatment. Several HSS interventions, based on the Effective Practice and Organisation of Care (EPOC) framework [16], were selected to address the contextual barriers and support the delivery of the HSS programme.

During the pilot evaluation phase of the study, three healthcare facilities were purposively selected to pilot the delivery of the HSS Programme. HSS interventions that were selected to support the delivery of the HSS programme components include training, group care, role expansion, audit and feedback, individual level care, referral systems, task sharing, delivery of individual level care, change to the healthcare environment and performance monitoring (Table 1). Data collection methods consist of a quantitative patient survey, observation of health promotion talks, patient file reviews, documentation reviews, assessment of counselling competence, and qualitative interviews with perinatal women and healthcare workers.

**Table 1:** Overview of the contextual barriers , HSS programme components, HSS interventions and data collection methods

Contextual Barriers	HSS programme components	HSS interventions	Collection methods
Poor patient knowledge and health seeking behaviour; high levels of stigma	**Health promotion and awareness raising** - delivery of health promotion and awareness raising talks • Provider – lay healthcare workers • Recipient – perinatal women • Place – waiting areas at MOU and BANC clinics • Time – in the morning • Frequency – daily • Tools – ASSET provided flipchart	**Training** – to equip healthcare workers with the knowledge, skills and tools to deliver the talks **Health education** – to provide pregnant women with health information **Delivery of group care** – to deliver talks to groups of women **Role expansion** – to task healthcare workers with delivering the talks in addition to their usual responsibilities **Audit and feedback** – to assess and discuss the delivery of talks with healthcare workers involved	**Qualitative interviews** with healthcare workers **Observation** of the health promotion and awareness talks and **completion of a checklist** used to capture healthcare worker competency, adherence to the structure, and environmental factors such as noise, lighting and size of area **Patient survey** – a self-administered survey questionnaire, completed during baseline and after the talks
Low levels of detection	**Detection** - of symptoms of CMDs and domestic violence in pregnant women as part of routine antenatal care • Provider – antenatal care nurses • Recipient – pregnant women • Place – at MOU and BANC clinics • Time – during routine consultations • Frequency – at every antenatal visit • Tools – Maternity Case Record (MCR) [17] and PACK guide [18]	**Training** – to equip healthcare workers with the knowledge and skills to detect women with CMDs and domestic violence **Delivery of individual-level care** – to detect women with symptoms of CMDs and domestic violence during private consultations **Audit and feedback** – to assess and discuss screening rates with healthcare workers involved	**Qualitative interviews** with pregnant women and ANC nurses **Documentation review** - review of patient files and documents used to record detection rates
Poor linkages to care	**Referral** - of pregnant women with symptoms of CMDs and domestic violence • Provider – antenatal care nurses • Recipient – pregnant women with CMDs or domestic violence • Place – at MOU and BANC clinics • Time – during routine consultations • Frequency – when a woman screens positive and consents to counselling • Tools – Referral form – Section A	**Training** – to equip ANC nurses with the tools and processes to link pregnant women who screen positive to care **Referral systems** – development of standardised referral pathways **Audit and feedback** – to assess and discuss referral rates with healthcare workers involved	**Qualitative interviews** with pregnant women and ANC nurses **Documentation review** - review of registers and referral forms used during the referral process
Limited availability of treatment	**Treatment** - provision of counselling sessions to perinatal women with symptoms of CMDs and domestic violence • Provider – community health workers • Recipient – perinatal women with symptoms of CMDs or domestic violence • Place – in patients’ homes • Time – as agreed by CHW and patient • Frequency – 3 sessions • Tools – Referral feedback – Section B	**Training –** to equip CHWs and OTLs with the knowledge, skills and tools to deliver psychological counselling **Task-sharing** – to task CHWs with delivering a psychological counselling program **Delivery of individual-level care** – to provide women with symptoms of CMDs and domestic violence with private psychological counselling sessions **Change to healthcare environment** – to provide psychological counselling in women’s homes or at off-site venues **Audit and feedback** – to assess and discuss counselling rates with CHWs and OTLs **Performance monitoring** – for OTLs to monitor the delivery of counselling sessions by CHWs	**Qualitative interviews** with pregnant women, OTLs and CHWs **Documentation review –** weekly reports on counselling progress

Implementation outcomes, as described by Proctor et al. [12] were selected to assess the delivery of each of the HSS programme components and included acceptability (satisfaction with the content and delivery of the component), appropriateness (perceived fit, usefulness and relevance of the component), feasibility (suitability of the component for everyday use), adoption (uptake, utilization and initial implementation of the component), fidelity of delivery (delivery of the component as intended) and fidelity of receipt [19] (receival of the component as intended). A cascaded training model was used to equip healthcare workers with the knowledge, skills and tools to deliver the HSS programme components. Several cadres of healthcare workers were trained as Master Trainers and tasked with cascading the training to facility- and community-based healthcare workers to deliver the HSS programme components. Further details are described in the pilot study protocol paper [11].

### Participants

#### Healthcare worker participants

Based on the roles that healthcare workers performed, and the Western Cape Department of Health’s (WC DoH) strategic plans, senior managers within the WC DoH identified a range of healthcare workers to be trained as Master trainers and as implementers of the intervention (Table 2). Facility-based psychiatric nurses were trained and tasked with cascading the training to facility-based health promotion officers and other lay healthcare workers to deliver the health promotion and awareness raising talks. The Practical Approach to Care Kit (PACK) - Primary Care Guide for the Adult (a clinical decision support tool for primary care workers) [18] was used as the foundation for the detection and referral component. Facility PACK trainers were tasked with cascading training to facility-based antenatal care (ANC) nurses who were responsible for detecting women with symptoms of CMDs and domestic violence and linking them to care. Community-based services (CBS) trainers from each of the sub-districts received the master training and were tasked with training CHWs (to provide psychological counselling to women with symptoms of CMDs and domestic violence) and OTLS (who supervised and supported CHWs). Purposively selected facility- and community-based healthcare workers involved in delivering the intervention participated in qualitative interviews and focus group discussions. All healthcare workers involved in the study provided written, informed consent. Further details are described in the pilot study protocol paper [11].

**Table 2:** Healthcare workers involved in the training and delivery of the HSS programme components

HSS Programme components	Healthcare workers trained as Master Trainers	Healthcare workers trained to deliver the HSS components
Awareness raising	Facility-based psychiatric nurses	Facility-based health promotion officers and other lay healthcare workers
Detection and referral	Facility-based Practical Approach to Care Kit (PACK) trainers	Antenatal care (ANC) nurses
Counselling	Community-based services (CBS) trainers	Community-based Community Health Workers (CHWs) and Outreach Team Leaders (OTLs)
Supervision of CHWs	CBS trainers	OTLs

#### Pregnant participants

Pregnant women, irrespective of gestational age, attending the selected MOUs and BANC clinics for routine antenatal care during the data collection period were invited to participate in a patient survey and/or qualitative interviews. All pregnant participants provided written, informed consent after receiving an information sheet and having the study verbally explained to them. Participants were informed that they were free to withdraw from the study without consequence.

### Data collection

The HSS programme was delivered between April and July 2021. Several data collection methods were used to evaluate the implementation outcomes of the four HSS programme components between April and October 2021.

#### Health promotion and awareness raising component

Data were collected using a patient survey, observation checklist and key informant interviews with healthcare workers. For the patient survey, pregnant women attending healthcare facilities were invited to complete a self-administered, bespoke, quantitative questionnaire containing socio-demographic questions, as well as questions on their knowledge, attitudes and health seeking behaviour with regards to depression, anxiety and domestic violence. The survey questionnaire was available in women’s home languages and was completed by (1) pregnant women who were not exposed to the health promotion and awareness raising talk (baseline), and (2) women who had just heard the talk (follow-up). While the talks were being delivered, research team fieldworkers observed the talks and completed an observation checklist to capture details of the talk such as language, length of time, information presented, number of women listening to the talk and environmental factors. Healthcare workers involved in the delivery of the talks were also invited to participate in qualitative interviews, using semi-structured interview guides.

#### Detection component

Data were collected using documentation reviews, key informant interviews and focus group discussions. The documentation review consisted of research team fieldworkers capturing the outcome of women’s mental health screening questionnaires found in their maternity case records (MCR) - the national stationery used to capture all aspects of the pregnancy care - as well as reviewing the patient registers completed by nurses to capture the total number screened, and number screened positive each day. In addition, several key informant interviews and focus group discussions were conducted with healthcare workers involved in the detection component as well as key informant interviews with pregnant women attending the healthcare facilities for routine antenatal care.

#### Referral component

Data were collected using documentation reviews, key informant interviews and focus group discussions. The documentation review consisted of reviewing the referral forms completed by ANC nurses for patients who agreed to home-based counselling, as well as reviewing the patient registers used to capture the number of women offered a referral and the number who agreed to the referral each day. In addition, focus group discussions were conducted with healthcare workers involved in the referral component and key informant interviews with pregnant women attending the healthcare facilities for routine antenatal care.

#### Treatment component

Data were collected using focus group discussions with healthcare workers involved in the treatment component and key informant interviews with pregnant women who were referred for home-based counselling. In addition, OTLs completed a bespoke checklist (during observation of counselling sessions) to assess CHWs fidelity to the counselling steps and structure. OTLs also completed weekly, written reports on the counselling stage and progress of all women whose referrals they were managing.

### Data analysis

Mixed methods were used to analyse the data collected. Qualitative interviews were transcribed by bilingual speakers. Transcripts were analysed by three researchers – two researchers analysed the same 5% of the transcripts, while a third researcher resolved any differences. Thereafter, all three researchers analysed the remaining transcripts. A framework analysis approach [20] was used. Development of the initial codes were guided by the interview topic guides. Themes not captured by the initial coding were identified through extensive reading of the transcripts.

Data collected during the documentation review were captured in Excel and are presented in numbers and proportions. The patient surveys were captured on Redcap by the research team fieldworkers and exported to STATA/SE statistical software package version 15.1 (StataCorp., College Station, TX, USA) for analyses. Data are presented as numbers and proportions, and associations measured using Chi-squared tests for categorical outcomes.

### Ethical approval

Research was conducted according to the principles of the Declaration of Helsinki. Ethical approval for the study was obtained from the Human Research Ethics Committee at the University of Cape Town (Ref No: 139/2018) and from the Psychiatry, Nursing and Midwifery Research Ethics Subcommittee at King’s College London (Ref No: 17/18-7807). The WC DoH approved the use of the research sites (WC_201911_004). All participants provided informed consent after receiving an information sheet and having the procedure verbally explained to them. Participants were informed of their right to withdraw from the study at any time without consequences.

## Results

Between March and September 2021, 139 perinatal women participated in key informant interviews. The majority were pregnant [*n*=125 (90%)], in their third trimester of pregnancy [*n*=74 (53%)] and spoke IsiXhosa [*n*=78 (56%)]. Eleven (*n*=11) key informant interviews were conducted with facility-based healthcare workers between March and May 2021. Two MOU Managers, seven ANC nurses and two health promotion officers were interviewed. Between June and August 2021, nine focus group discussions were conducted with ANC nurses (*n*=3) and OTLs (*n*=6).

### Training

Master trainers received their training in January 2020 and were tasked with cascading the training to the relevant healthcare workers in February 2020 (Table 2). As a result of the COVID-19 pandemic in March 2020, many of the healthcare workers who had received training, were no longer available in 2021 when delivery of the HSS components were assessed. Several new and refresher trainings were held with all healthcare workers involved between March and June 2021. Further details are available in the protocol paper [11].

#### Health promotion and awareness raising

Healthcare workers reported that their one-day training in 2020 was adequate and had equipped them with the skills needed to deliver the talks. One healthcare worker explained that the training had taught her to identify “*the person who is behaving out of normal character*” [G006H], which helped her identify women who needed to be referred to the nurse.

#### Detection and referral

Facility PACK trainers were first trained as master trainers. However, as a result of the pandemic, the facility PACK trainers were no longer available in 2021 and ASSET team members had to provide refresher training to some ANC nurses and completely new training to others. The modules that were intended to be delivered weekly over a four-week period, had to be condensed and delivered in a four-hour training session, with minimal time for exercises. Some ANC nurses reported that the training was very informative while others explained that they did not use the PACK guide in the antenatal clinic. Instead, nurses were trained to follow the BANC guidelines. At one MOU, the nurses reported that the training was too long, as they had been screening pregnant women (only those attending the healthcare facility for their first antenatal care visit) for symptoms of CMDs using the mental health screening questionnaire (released by the DoH after completion of the pre-implementation phase of the study, in an updated version of the MCR).

#### Counselling and supervision

In addition to the original counselling and supervision training in 2020, several additional trainings were delivered by the master trainers who were originally trained. While some OTLs reported that the training was adequate and had equipped the CHWs with the necessary skills, many felt that they needed more time for role-playing and practicing how to deliver the three sessions. One OTL reported that the CHWs she supervised *“… didn’t have the confidence. They didn’t feel adequately trained*” [R02FGD]. She suggested that the training, which was delivered over three days, would work better if it was delivered over a longer period, with time in between where CHWs could practice the skills they were taught before learning the next skill.

### HSS programme components

The results are reported for each of the HSS programme components using the following implementation outcomes: acceptability, appropriateness, feasibility, adoption, fidelity of delivery, and fidelity of receipt (Table 3).

**Table 3:** Implementation outcomes for each of the HSS Programme components

	Health promotion and awareness raising	Detection	Referral	Treatment
**Acceptability** – satisfaction with the content and delivery of component	Acceptable to healthcare workers	Acceptable to some, but not all pregnant women	Acceptable to be linked to care, but not to be visited at their homes	Acceptable to pregnant women, but not to all CHWs
**Appropriateness** – usefulness, relevance, suitability of component	Appropriate to healthcare workers	Appropriate to healthcare workers and pregnant women	Appropriate to healthcare workers	Appropriate to healthcare workers and pregnant women
**Feasibility** – suitability of component for routine implementation	Feasible to some extent	Not feasible using ANC nurses	Not feasible to be contacted at home	Not feasible to be counselled by CHWs
**Adoption** – uptake, utilization and initial implementation of the component	Poor adoption	Poor adoption	Poor adoption	Poor adoption
**Fidelity of delivery** – delivery of the component as intended	Poor fidelity	Poor fidelity	Poor fidelity	Poor fidelity
**Fidelity of receipt** – user understanding and performance resulting from receipt of the component	Improved intended health seeking behaviour	Improved detection rates	Improved referral rates	Decreased levels of distress

#### HSS programme component 1: Health promotion and awareness raising

This health promotion and awareness raising component involved health promotion officers, or other lay healthcare workers, delivering a 5–7-minute presentation to small groups of women in the waiting areas, using a flipchart as a visual aid. The presentation included information on the signs, symptoms, risk factors and consequences of depression, anxiety and experiences of domestic violence as well as to inform women about the mental health screening delivered by the ANC nurses and the home-based counselling delivered by CHWs for women with symptoms of CMDs or domestic violence. As very few talks were delivered towards the end of the study when the qualitative interviews took place, we were unable to assess the acceptability, appropriateness and feasibility from a user perspective.

##### Acceptability – satisfaction with the content and delivery of the health promotion and awareness raising component

Healthcare workers considered the provision of the health promotion and awareness raising talks to be acceptable. An ANC nurse explained that the pregnant women “*enjoyed the talks*” [R01FGD]. In addition, the content of the talks played an important role in creating awareness of the symptoms of CMD. An ANC nurse described how women would disclose their feelings “*as soon as they come in* (to the consultation room)” by saying “*I heard the talk and I do feel like that Sister*” [R01FGD].

##### Appropriateness – perceived fit, usefulness and relevance of the health promotion and awareness raising component

Healthcare workers felt that the talks were especially useful in alerting pregnant women to the mental health screening questions that would be asked, as well as the availability of counselling by CHWs for those who screened positive. A healthcare worker explained that the talks were “*helpful to the patients, because the patients now they are willing to talk to us*” [G006H]. She elaborated by saying that “*they* (pregnant women) *know where to get help if they have a problem, they know who they can talk to*” and that the talks should be “*introduced at other clinics* (healthcare facilities) *that see pregnant women*”. A health promotion officer explained that, before delivery of the talks, pregnant women “*didn’t know that when you come to the clinic, you come for many things* (including mental health counselling) *… they thought you only come for pregnancy*” [G001H].

##### Feasibility – suitability of the health promotion and awareness raising component for everyday use

While the content of the talks was considered suitable, provider-related issues impacted its suitability for everyday use. Following delivery of the talks, some women would seek out the healthcare worker who had delivered the talk to discuss the problems they were experiencing, leaving the healthcare worker feeling distressed and in need of support. A health promotion officer reflected on her experiences by saying “*after you speak to the client, you sit down and reflect on that problem. It sometimes leaves you feeling depressed”* [G001H].

##### Adoption – uptake, utilization and initial implementation of the health promotion and awareness raising component

The talks were not delivered regularly. At facilities without a dedicated health promotion officer, those who were trained reported that their other responsibilities were often time sensitive, which made it difficult to deliver the talks in the morning when the perinatal women were waiting for their consultations with the midwives. Healthcare workers also rotated into different departments, which resulted in those who were trained to deliver the talks, being unavailable a few months after their training. At the BANC clinic, the breastfeeding counsellor initially delivered a talk 3-4 days per week, while the enrolled nurse at the MOU delivered the talks daily. At both facilities the healthcare workers were unable to provide the talks from August when they were rotated to a different department. At the MOU with a dedicated health promotion officer, the talks were delivered approximately twice per week. On the other days, the health promotion officer chose to present other health-related information such as the stages of pregnancy, HIV testing, hygiene, and breastfeeding.

##### Fidelity of delivery – delivery of the health promotion and awareness raising component as intended

The talks were not always delivered as intended. Only one facility had a dedicated health promotion officer. At the other facilities, other lay healthcare workers were identified to provide the talks. At the BANC clinic, a breastfeeding counsellor was trained to provide the talks. She never used the flipchart, and only spoke about depression and domestic violence. At one of the MOUs, an enrolled nurse always used the flipchart and delivered the talk as intended. The second MOU had a dedicated health promotion officer who was trained. She chose not to use the flipchart, and in most cases spoke only about depression.

##### Fidelity of receipt – receival of the health promotion and awareness raising component improved knowledge and practices as intended

Five hundred and forty-five (*n*=545) women completed the knowledge and practices survey in the two weeks prior to the delivery of the HSS programme (baseline) and 650 women completed the survey after an awareness raising talk (follow-up). We found an improvement in health seeking behaviour after the talks (Table 4). Significantly more women were prepared to disclose feelings of depression or anxiety to family members (83% vs. 77%; *p*=0.020); and to disclose feelings of depression (82% vs. 76%; *p*=0.011) and anxiety (83% vs. 77%; *p*=0.009) to healthcare workers after the talks, compared to before the HSS programme was delivered. There was no significant change in the proportion of women who were prepared to disclose experiences of domestic violence to a family member (*p*=0.504) or healthcare worker (*p*=0.642) after the talks, compared to before the HSS programme was delivered.

**Table 4:** Knowledge, attitudes and health seeking behaviour of pregnant women

	Baseline ***N***=583	Follow-up ***N***=652	*P*-value*
**Knowledge**			
Correct answer to symptoms of depression	305 (56.0)	362 (55.7)	0.925
Correct answer to symptoms of anxiety	281 (51.6)	335 (51.5)	0.994
Correct understanding of domestic violence	425 (72.9)	502 (77.0)	0.138
Believes that depression and anxiety can be helped with medication	294 (53.9)	376 (57.9)	0.176
Believes that depression and anxiety can be helped with counselling	427 (78.4)	500 (76.9)	0.556
Believes that women who are abused can get out of abusive relationships	438 (80.4)	517 (79.5)	0.722
**Attitudes**			
Uses the word ‘crazy’ to describe people with mental health disorders	44 (8.1)	55 (8.5)	0.808
Thinks that domestic violence is acceptable under certain circumstances	76 (13.9)	120 (18.5)	**0.036**
**Health seeking behaviour**			
Prepared to disclose feelings of depression or anxiety to family	420 (77.1)	536 (82.5)	**0.020**
Prepared to disclose feelings of depression to a staff member	415 (76.2)	534 (82.2)	**0.011**
Prepared to disclose feelings of anxiety or stress to a staff member	422 (77.4)	542 (83.4)	**0.009**
Prepared to disclose domestic violence to family	463 (84.9)	543 (83.5)	0.504
Prepared to seek help for domestic violence from the police or a social worker	477 (87.5)	563 (86.6)	0.642

*Chi-squared test

#### HSS programme component 2: Detection

Detecting pregnant women with CMD and domestic violence involved an ANC nurse administering a brief screening questionnaire (available in the MCR [17]), using a patient-centred approach, to all pregnant women during their routine consultations. Women who screened positive underwent further assessment using the Practical Approach to Care Kit (PACK) - Primary Care Guide for the Adult [18].

##### Acceptability – satisfaction with the content and delivery of the detection component

While many of the pregnant women interviewed expressed acceptability of the screening process, some women were reticent about revealing how they felt to an ANC nurse. One pregnant woman explained that the ANC nurse “*did not ask anything that could start the conversation, she concentrated on the examination”* [N045P], while another woman explained that “*it’s just not easy talking to a nurse*” [N047P]. An MOU manager explained that “*Unfortunately, we as nurses have a bad name out there. Not everybody feels comfortable speaking up – to voice out what they want to say, they will choose certain people*” [G004H].

Other women were concerned about the stigma of disclosing domestic violence to a nurse. A pregnant woman who was experiencing domestic violence explained that she felt ambivalent about disclosing her experiences because “*when you tell the truth, people might judge you*” [G045P].

##### Appropriateness – perceived fit, usefulness and relevance of the detection component

All healthcare workers interviewed reported that the screening process was useful for detecting women with symptoms of CMD and domestic violence. One healthcare worker explained that the screening was “*something that we’ve needed for quite some time in the maternal aspect, because it’s certain things that can be easily ignored*” [G005H]. One ANC nurse commented on the importance of making eye contact (which formed part of the detection component) with a patient when administering the mental health questionnaire by saying “*you have to make time to look … in the eyes. If the patient doesn't make eye contact, you know there is something going on*” [RGF01]. Many pregnant women confirmed that the mental health questionnaire administered by the ANC nurses gave them the opportunity to express how they were feeling, which they would not have disclosed if they had not been specifically asked.

##### Feasibility – suitability of the detection component for everyday use

There were several challenges observed related to the feasibility of the detection process. The proportion of women who were detected with symptoms of CMD when a research team fieldworker did the screening was significantly more than when the ANC nurses did the screening [474/1198 (28%) vs. 190/9000 (2%)], even though the fieldworkers screened fewer women than the nurses each day (each facility had one fieldworker versus several nurses). An ANC nurse explained that when she screens patients they would say “*I’m fine sister, I don’t have any problems, I’m fine*” [GFG01], yet the same patient would screen positive when the fieldworker did the screening (using the same questions).

One ANC nurse explained that patients do not want to disclose their experiences in the consultation rooms as the cubicles offer little privacy – “*it’s only the curtains so the patient is sitting here, she can hear what is going on, on the other side*” [RFG01].

Several ANC nurses reported that patients’ disclosures during the screening process were sometimes difficult to listen to. One ANC nurse said that “*it’s not that we don’t want to do these things* (screening)*, we are doing it, but sometimes it gets too much*” [G004H]. Another ANC nurse elaborated by saying “*we are unable to cope because we are facing our own problems”* [GFG01]. A third ANC nurse described how a patients’ disclosure had affected her by saying “*the patient was very emotional, and I was seeing to the patient, so I also broke down*” [GFG01].

Many healthcare workers involved in the screening process were unhappy about the increased workload. Administering the screening questions, completing additional documents and calming patients who became teary during the screening process added additional time to their already busy schedules. One healthcare worker explained that “*we are already ticking a lot of things… and filling in a lot of forms. And it’s taking up a lot of our time*” [G004H].

##### Adoption – uptake, utilization and initial implementation of the detection component

The screening component of the HSS programme was delivered inconsistently. ANC nurses at one facility chose to only screen patients at their first antenatal clinic visit, while ANC nurses at the other healthcare facilities skipped the screening process during busy times. One ANC nurses admitted that “*we don’t do it (screening) routinely*” [G002H]. As a result, nurses reported only being able to screen 30-40% of the women attending antenatal clinics each day.

##### Fidelity of delivery – delivery of the detection component as intended

The majority of nurses referred women for counselling after screening positive on the brief screening questionnaire instead of using the PACK guide to assess the severity of symptoms or exclude other possible reasons for their symptoms (such as bereavement, medication side effects, or substance abuse) before making the referral. ANC nurses reported that they did not feel comfortable or confident using the PACK guide as only a few of them had received prior PACK training. An OTL described how high-risk cases were inappropriately referred to the NPO by saying “*there are cases where a mom is referred to us but their situation is beyond our capabilities… There is a lot of cases that needs more of the professional help than what we can give, abuse is one to mention*” [GFG02].

##### Fidelity of receipt – receival of the detection component improved detection rates as intended

A review of the mental health screening questionnaire in the MCR (completed by ANC nurses) was used to assess changes in detection rates (Table 5). During the delivery of the HSS programme, both the proportion of women screened [611 (75%) vs. 496 (60%); *p*<0.001], as well as the proportion of women detected with CMD [42 (7%) vs. 17 (3%); *p*=0.011] improved significantly.

**Table 5:** Detection of CMD before and during delivery of the HSS programme

	Before delivery of HSS programme ***n***=831	During delivery of HSS programme ***n***=819	***P***-value*
Mental health screening questionnaire administered (*n*=1650)	496 (59.7)	611 (74.6)	<0.001
Screened positive	17 (3.4)	42 (6.9)	0.011
Screened negative	479 (96.6)	569 (93.1)	

*Chi-squared test

**≥2 on the mental health screening questionnaire

#### HSS programme component 3: Referral

Linkage to care involved: (1) ANC nurses completing a referral form for women who screened positive and agreed to the counselling; (2) emailing the referral form to the community-based services coordinator, who reviewed and forwarded the referral form to the relevant NPOs or when the physical referral form was collected from the healthcare facility and delivered to the NPO office; (3) assigning the patient to an OTL and CHW based on the woman’s address (OTLs and CHWS serviced a pre-defined area);and (4) an OTL or CHW making telephonic or physical contact with the referred woman to arrange counselling.

##### Acceptability – satisfaction with the content and delivery of the referral component

Many pregnant women who were interviewed at the healthcare facilities indicated that they would be happy to be referred to a CHW, social worker or mental health nurse if they required support. However, several women did not want anyone to visit them at home and indicated that unless the counselling took place at the healthcare facility, they would not accept the referral. Some CHWs had difficulty articulating the reason for the visit following a referral, especially when using one of the local languages (Afrikaans and IsiXhosa), which resulted in women being dissatisfied with the referral. One pregnant woman described the CHWs IsiXhosa reason for the visit by saying “*someone did come to my house, but she said they were helping crazy people. So I told them I’m not crazy… so they never came again*” [N047P].

Some women provided incorrect contact information because (1) they feared being turned away from their facility of choice because they did not reside in the immediate vicinity, or (2) they agreed to the counselling to please the nurse and didn’t fully understand what they were agreeing to. One OTL recounted her experience with a pregnant woman who had been referred by saying “*Sometimes they will tell you - I just signed that paper there to please the sister at the MOU just to get her off my back*” [RFG02].

##### Appropriateness – perceived fit, usefulness and relevance of the referral component

Healthcare workers based at the MOUs were happy to email their referrals to the community-based service office for dissemination to the NPOs. The process was familiar to all involved as the same process was used to refer other service users requiring community or home-based support. Since the BANC clinic only had one supporting NPO, those involved found the collection of physical forms a more suitable method.

##### Feasibility – suitability of the referral component for everyday use

While ANC nurses were happy to complete the referral forms, administrators and managers who were tasked with ensuring that the forms were emailed to the community-based service office reported that the increased workload was sometimes quite difficult to manage. One manager explained her dilemma by saying “*sometimes I do forget to scan the referrals, especially Fridays because I am not here Saturday and Sunday, … but my aim was to always try and push and make sure that people are being seen as soon as possible*” [G004H].

##### Adoption – uptake, utilization and initial implementation of the referral component

Many women returned to the facility weeks after being referred for counselling, without anyone having made contact with them. In some instances, by the time CHWs were able to contact a patient, their feelings of distress had already been resolved, or they had accessed support elsewhere. One ANC nurse explained that patients returned to the facility saying “*I am still having a problem and they didn’t get hold of me*” [NFG01]. The delay or absence in contacting women occurred for several reasons such as: (1) incomplete or illegible referral forms; (2) incorrect contact details on referral forms; (3) delays in emailing the referral forms to the community-based services coordinator;(4) delays in a community-based services coordinator accessing the referral forms and forwarding them to the relevant NPO;and (5) delays in OTLs contacting patients. One OTL explained that “*sometimes it’s difficult as some patients give wrong addresses, phone numbers that don’t exist or they will say they don’t have a number, it’s not easy to trace them*” [GFG02].

##### Fidelity of delivery – delivery of the referral component as intended

While a referral form was always completed for patients who required a referral, the forms often contained incorrect contact details, making it difficult to find the women. Many women did not own a cell phone, so were not able to provide their contact details or provide the contact number of a friend, relative or neighbour. In addition, seventeen (9%) of the women were incorrectly referred for home-based counselling as they were high risk cases that needed specialised support. These women were referred back to the facility, where they received a referral to see a mental health nurse or social worker for further support.

##### Fidelity of receipt – receival of the referral component as improved referral rates as intended

The referral system was partially effective at linking patients to care**.** Of the 198 women who were referred for counselling, 59% were contactable. Illegible, incorrect or outdated contact details were responsible for being unable to locate 41% of those referred.

#### HSS programme component 4: Treatment

The treatment component consisted of three structured counselling sessions, using a problem-solving approach, delivered by CHWs in patients’ homes or at the healthcare facility, while being supervised and supported by OTLs [21].

##### Acceptability – satisfaction with the content and delivery of the counselling component

All women who experienced one or more counselling sessions (*n*=39) reported being satisfied with the counselling and that it had helped them cope when they were feeling distressed. One pregnant woman explained how counselling had helped her by saying *“before I attended the counselling, I was crying a lot. Everything was hard for me. But since I spoke to them* (CHWs)*, at least it went down, it was good to talk to them*” [G044P]. Another pregnant woman described her feeling of comfort while being counselled by saying “*when they are with me, it’s like I was with my sisters*” [G043P]. While some CHWs were happy to deliver the counselling sessions, others were uncomfortable supporting women that came from the same communities that they lived in and were experiencing the same problems they were experiencing. One OTL explained that some of the CHWs “*don’t think that this is where they want to be – to give counselling…so we can’t force them*” [R02FGD].

##### Appropriateness – perceived fit, usefulness and relevance of the counselling component

Several OTLs and CHWs reported on the usefulness of the counselling content. Some mentioned that they were able to use the counselling skills for their other patients, not just the pregnant women who were referred for counselling. One OTL commented on how it had “*helped us look at the mental state and not just going to see the mums and babies*” [R004H]. Many of the women who received at least one counselling session expressed how useful the counselling had been. One pregnant woman described how burdened she felt before she received counselling, and that the counselling “*was actually good*” [*G045P*] . She explained that after she started the counselling, she felt “*at ease a bit – not totally, but I felt good. I felt like I can talk to them* (the CHW and OTL) *freely without them judging me. They were very welcoming, very supportive and very understanding at the same time*”.

##### Feasibility – suitability of the counselling component for everyday use

Several issues were raised regarding the feasibility of the counselling component. Both patients and healthcare workers felt that CHWs were not the right cadre of staff to provide counselling. As CHWs lived in the same community in which they worked, patients were concerned about confidentiality. In addition, OTLs were concerned about the capability of CHWs as they were lay healthcare workers with no previous mental health training or experience. An OTL explained that CHWs “*have never dealt with anything mental before… they’ve got no experience; even their home-based care training, some of them it’s so minimal … there are huge problems there with using CHWs in the communities”* [RFG02].

Using patients’ homes for counselling was also problematic, as many women lived in small homes or informal settlements with large household numbers. It was often difficult to find a private space to deliver the counselling. One pregnant woman explained that “*We live in a shack, so when you talk in your house, someone can hear you from next door, so it’s not private enough*” [G045P]. In addition, the some women were concerned that other household members and neighbours would be curious about the CHW visits, as these visits were historically connected to patients with HIV or TB. Some women voiced their concern that others would assume that they were receiving treatment for HIV or TB.

Both CHWs and OTLs reported feeling distressed after hearing patients’ experiences of distress, especially when they were describing domestic violence. One OTL described her difficulty by saying “*I struggled to shut the visuals in my mind. I couldn’t sleep that night*” [GFG02].

##### Adoption – uptake, utilization and initial implementation of the counselling component

Between April and July 2021, 198 pregnant women were referred for home-based counselling (Table 6). Only 20 (10%) of those referred completed all three counselling sessions and reported feeling better able to cope. An additional 19 (10%) felt better after one or two sessions while 13 (7%) reported feeling better without counselling. Many women were either not found [82(41%)] or no longer interested in receiving counselling [35 (18%)] once they knew what it entailed. Some women misunderstood the referral and thought that the CHWs would be bringing them money, food or clothes for the baby.

**Table 6:** Outcomes of women referred for home-based counselling

Outcomes of women referred for counselling	***n***=198 n (%)
**Received counselling**	
Completed all counselling sessions	20 (10.1)
Completed <3 counselling sessions	19 (9.6)
**Did not receive any counselling**	
Referred back to facility for specialised support	17 (8.6)
Felt better by the time they were contacted	13 (6.6)
No longer interested in counselling	35 (17.7)
Could not be found	82 (41.4)
Moved and was no longer living in the area supported by an NPO linked to the study	9 (4.5)
Unavailable during working hours	3 (1.5)

##### Fidelity of delivery – delivery of the counselling component as intended

Many CHWs had difficulty delivering the counselling sessions as intended. Even though OTLs spent time preparing the CHW for a counselling session, they often became overwhelmed during the counselling session and did not follow the structured process. An OTL described how CHWs were “*allowing patients to go off-topic and load it with all sorts of things, but not following the steps*” [RFG02]. A pregnant woman who received counselling confirmed the OTLs’ views when she explained that the CHW tasked with providing the counselling had “*asked me to pray about the situation whenever I feel like I’m alone or I don’t have someone to talk to. I should just talk to myself about the whole thing as if I’m with someone*” [G047P]. Another pregnant woman explained that the CHW had given her a book to “*read to forget*” and had advised her to speak to her pastor to “*help her cope*” [R039P]. Yet, the few OTLs who evaluated the CHWs fidelity to the counselling steps and structure using a checklist, reported that the steps were done partially or done well. Further comments on the evaluation forms referred to the CHWs interest in helping the patients, their good listening skills and their efforts to reassure patients of the confidentiality of the counselling sessions.

##### Fidelity of receipt – receival of the counselling component decreased symptoms of distress as intended

Even though the counselling sessions were not delivered as intended, all the pregnant women who received counselling reported feeling better after speaking to a CHW. A 20-year-old who was pregnant with twins explained that she “*could open up to someone I don’t really know and they made me feel comfortable*” [R037P]. Many women appreciated having someone that checked on them and cared about how they were feeling. One woman explained that “*they just come to see what I’m doing … and to ask me about my pregnancy and when I’m due*” [R034P], while another woman explained that she found it helpful to “*talk to someone else that can relate or have an understanding*” [R033P].

## Discussion

Using several data collection methods in a pilot trial, we were able to evaluate the implementation outcomes of four HSS programme components, namely health promotion and awareness raising, detection, referral and treatment, for perinatal women with CMD or experiences of domestic violence in Cape Town, South Africa. In this study healthcare workers found the delivery and content of the health promotion and awareness raising talks to be both acceptable and appropriate. Even though the feasibility, fidelity of delivery and adoption of the talks were poor, we found significant improvement in patients’ health seeking behaviour, suggesting good fidelity of receipt. While the detection and referral components were considered appropriate and acceptable to some pregnant women, these components performed poorly with regards to feasibility, fidelity of delivery and adoption. However, we demonstrated a significant increase in both the proportion of women who were being screened, as well as the proportion who were detected with symptoms of CMD. Both the healthcare workers and pregnant women found the counselling component acceptable and appropriate. Despite the poor feasibility, fidelity of delivery, and adoption of the counselling component, women who received counselling reported feeling better able to cope with their situation, indicating good fidelity of receipt.

The first component of the HSS programme made use of health education (a strategy of health promotion) and role expansion to improve knowledge, decrease stigma and improve health seeking behaviour or perinatal women. At all three facilities, healthcare workers described the importance of delivering the talk regularly, yet very few talks were delivered during the evaluation period, and they were rarely delivered as intended. The health education approach has received some criticism, as it is expert-led, didactic, assumes that the target audience has little to no knowledge, and ignores the educational and social determinants affecting the audience’s use of the newly acquired knowledge [22]. Still, the delivery and content of the talks were acceptable to healthcare workers and pregnant women and was able to change women’s attitudes towards seeking help for mental health conditions. We chose to expand the role of health promotion officers to include a mental health awareness talk, as health promotion officers are facility-based CHWs, employed by the DoH at primary healthcare facilities, whose duties include health education [23]. However, delivery by health promotion officers was not feasible as many facilities did not have a dedicated health promotion officer, and even when one was available, the uptake and fidelity of delivery of the talks were poor.

Supervision is considered to be key to ensuring that healthcare workers have well-defined roles, are motivated and perform well [24]. As health promotion officers reported to the facility manager, and were given minimal supervision, it is not surprising that the talks were not delivered as or when intended. Evidence from a review of CHW supervision suggests that improving the quality of supervision is more important than increasing the frequency of supervision. Moreover, supportive supervision demonstrated improvement in healthcare worker performance and the quality of the care they provided [25].

The detection and referral components of the HSS programme were considered acceptable, yet we found many challenges relating to the feasibility of ANC nurses conducting the screening, as well as using a referral system whereby patients were contacted to arrange for a counselling session after leaving the healthcare facility. ANC nurses, whose primary role is to monitor the physical well-being of the mother and her pregnancy, found it difficult to incorporate the mental health screening and accompanying referral documents into their already busy schedules, resulting in poor fidelity of delivery and adoption of both the detection and referral components. This is consistent with previous research reporting that nurses working in the public sector in South Africa are overworked due to staff shortages and the need to provide integrated services as required by the South African DoH [26]. As in our study, other studies have also reported that overworked nurses produce illegible, inaccurate, and incomplete documentation, as multiple registers and documents have to be completed for each patient [27]. The incomplete and inaccurate completion of referral forms greatly impacted the OTLs’ and CHWs’ ability to trace patients, which contributed to the poor uptake of the treatment component.

Both ANC nurses and patients reported discomfort with the delivery of the detection component – nurses felt uncomfortable administering the questionnaire, and patients expressed reluctance to disclose sensitive information to nurses who appeared busy and uninterested. Again, these findings are not surprising as similar concerns were reported in other studies [28–30] as well as during the pre-implementation phase of the ASSET study [10]. While using digital technology such as mobile phone applications instead of verbal screening by nurses may improve the discomfort reported by patients and ANC nurses, inadequate access to the internet at healthcare facilities remains a challenge.

Similar to the detection and referral components, the delivery and content of the treatment component was acceptable and appropriate, and all women who received the counselling reported significant benefit – in keeping with a previous meta-analysis of the effectiveness of task sharing psychological treatments in low- and middle-income countries (LMIC) [31]. Yet delivery of the counselling by CHWs in patients’ homes was not feasible or acceptable, contributing to poor fidelity of delivery and adoption, consistent with findings from a task-shared psychosocial intervention for perinatal depression in Cape Town [32]. In LMIC, task-sharing has been proposed as a strategy for scaling up mental healthcare, and has been successfully implemented in several countries including South Africa [33–35]. However, in our study, patients reported mistrust of CHWs as well as lack of confidence in their abilities, as CHWs lived in the same community they worked in and were known to many patients. In addition, both CHWs and their supervisors had little confidence in their ability to deliver the counselling.

### Recommendations

Based on these findings, we recommend appointing a dedicated facility-based lay health worker (FB-LHW) at each facility. The FB-LHW should be trained and supported by Registered Counsellors. The role of the dedicated FB-LHW would consist of (1) delivering the health promotion and awareness talks daily, (2) screening all pregnant women attending MOUs and BANC clinics using the mental health screening questionnaire in the MCR, (3) providing counselling to those with mild/moderate symptoms of CMDs, at facilities, on the same day, and (4) referring patients with more severe symptoms to the Registered Counsellors for further assessment and care.

Our study found that if the talks are done well, they can be very effective in creating awareness and will likely lead to greater uptake of the counselling by those in need. By using the same person to deliver the talks and do the screening, training will be more sustainable, the correct information will be conveyed to the patients, patients will have more confidence in the FB-LHW abilities, and it will decrease the amount of time spent on psychoeducation during the counselling sessions, making the counselling sessions more efficient. Utilizing the same person to deliver the talks, screen, and counsel patients will foster a trusting relationship between the lay healthworker and the pregnant women, while providing counselling at the healthcare facility on the same day will eliminate the many issues related to referring and tracing women after they’ve left the healthcare facility, as well as delivering the counselling in women’s homes. If facility-based Registered Counsellors are responsible for training, supervising and supporting the FB-LHW, they will receive better training and emotional support, and will be more adequately supervised, especially if the job descriptions include key performance indicators which are regularly assessed.

### Limitations

Although our sample size is relatively large and we recruited until saturation, we delivered and evaluated the HSS programme in only three facilities. Our results may not be generalisable to other parts of South Africa, or even the Western Cape. We were not able to completely limit social desirability bias as the researchers who were known to some participants, conducted the key informant interviews and focus group discussions. We used a bespoke survey questionnaire to assess change in knowledge, attitudes and health seeking behaviour, and did not have a control group, making it difficult to attribute the changes observed to the health promotion and awareness talks. We were only able to assess the implementation outcomes over a four-month period. As behaviour change often takes a lot longer than four months, we may have observed better adoption and fidelity over time. The impact of COVID-19 on healthcare workers and patients could not be measured as healthcare workers were trained before the COVID-19 pandemic, but the HSS programme could only be implemented a year later.

## Conclusions

This study provides evidence of the acceptability, appropriateness, feasibility, adoption, fidelity of delivery and fidelity of receipt of a health promotion and awareness raising, detection, referral and treatment HSS programme, delivered by ANC nurses and lay healthcare workers for perinatal women with CMDs and experiences of domestic violence. Our findings highlight the acceptability and appropriateness of the programme components, while poor feasibility of the model resulted in poor fidelity and adoption. Finally, we recommend appointing dedicated, lay healthcare workers to deliver key programme components at healthcare facilities, on the same day as patients screen positive. Further research is required to evaluate the implementation of this model at scale.

## Data Availability

The data that support the findings of this study are available from Dr Zulfa Abrahams but restrictions apply to the availability of these data, which were used under license for the current study, and so are not publicly available. Data are however available from the authors upon reasonable request and with permission of Dr Zulfa Abrahams.

## Abbreviations

ANC: antenatal care
ASSET: Health Systems Stregthening in sub-Saharan Africa
FB-LHW: facility-based lay health worker
BANC: basic antenatal care
CBS: community-based services
CHW: community health worker
CMD: common mental disorders
EPOC: Effective Practice and Organisation of Care
HSS: Health systems strengthening
MOU: midwife obstetric unit
MCR: maternity case record
NPO: not-for-profit organisation
OTL: outreach team leader
PACK: practical approach to care kit
PHC: primary healthcare
LMIC: low- and middle-income country
